# Integrative computational immunogenomic profiling of cortisol‐secreting adrenocortical carcinoma

**DOI:** 10.1111/jcmm.16936

**Published:** 2021-10-19

**Authors:** Jordan J. Baechle, David N. Hanna, Konjeti R. Sekhar, Jeffrey C. Rathmell, W. Kimryn Rathmell, Naira Baregamian

**Affiliations:** ^1^ School of Medicine Meharry Medical College Nashville TN USA; ^2^ Division of Surgical Oncology & Endocrine Surgery Department of Surgery Vanderbilt University Medical Center Nashville TN USA; ^3^ Department of Pathology, Microbiology, and Immunology Vanderbilt University Medical Center Nashville TN USA; ^4^ Department of Medicine Vanderbilt University Medical Center Nashville TN USA

**Keywords:** adrenocortical carcinoma, cortisol secreting adrenocortical carcinoma, cushing's syndrome, immunometabolism, tumour immunology, tumour microenvironment

## Abstract

Adrenocortical carcinoma (ACC) is a rare but highly aggressive malignancy. Nearly half of ACC tumours overproduce and secrete adrenal steroids. Excess cortisol secretion, in particular, has been associated with poor prognosis among ACC patients. Furthermore, recent immunotherapy clinical trials have demonstrated significant immunoresistance among cortisol‐secreting ACC (CS‐ACC) patients when compared to their non‐cortisol‐secreting (nonCS‐ACC) counterparts. The immunosuppressive role of excess glucocorticoid therapies and hypersecretion is known; however, the impact of the cortisol hypersecretion on ACC tumour microenvironment (TME), immune expression profiles and immune cell responses remain largely undefined. In this study, we characterized the TME of ACC patients and compared the immunogenomic profiles of nonCS‐ACC and CS‐ACC tumours to assess the impact of differentially expressed genes (DEGs) by utilizing The Cancer Genome Atlas (TCGA) database. Immunogenomic comparison (CS‐ vs. nonCS‐ACC tumour TMEs) demonstrated an immunosuppressive expression profile with a direct impact on patient survival. We identified several primary prognostic indicators and potential targets within ACC tumour immune landscape. Differentially expressed immune genes with prognostic significance provide additional insight into the understanding of potential contributory mechanisms underlying failure of initial immunotherapeutic trials and poor prognosis of patients with CS‐ACC.

## 1 INTRODUCTION

Adrenocortical carcinoma (ACC) is among the rarest and most aggressive cancers. Although the current prognostication of patients with ACC primarily hinges on the presence or absence of metastases and tumour resectability, over a third of patients present with an advanced, unresectable disease.^1^, ^2^, ^3^, ^4^, ^5^, ^6^ Patients with fully resectable (R_0_) disease have a reported 5‐year survival rate of approximately 50%, whereas patients with the unresectable disease have a 5‐year survival rate near 0% and a median survival of shorter than 12 months.^4^, ^7^, ^8^ Although large collaborative studies have greatly enhanced the molecular characterization of ACCs^9^, ^10^ aside from the advent of mitotane therapy in the treatment of ACC since 1959, there has been little improvement in overall mortality over the past several decades.^11^, ^12^ Due to the limited therapeutic options for patients with unresectable ACC, several immunotherapies are currently under evaluation;^13^, ^14^, ^15^, ^16^ thus, having a comprehensive understanding of the ACC tumour microenvironment is important for guiding future therapeutic directions.

Nearly half of patients presenting with ACC have been shown to exhibit steroid hormone hypersecretion with excess cortisol secretion being the most predominant hormone and often considered a strong risk factor for poor prognosis.^6^, ^17^ Glucocorticoids, including cortisol, are small lipid hormones produced by the adrenal glands that exert their effects through glucocorticoid receptors modulating gene expression to perform a variety of functions, including arresting immune cell growth and maturation, inhibiting activation signalling and inducing lymphocyte apoptosis.^18^, ^19^ Glucocorticoids have proven so effective in this role that they are the cornerstone of treatment for many hypersensitive immune reactions and autoimmune diseases.^20^, ^21^ However, the immunosuppressive effects of excess glucocorticoid therapy and hypersecretion have also been shown to hinder the immune system's capacity to ward off infections and malignancy and have been associated with a variety of other effects, including muscle wasting, osteoporosis and metabolic derangements.^22^, ^23^ A recent in vivo study by Landwehr et al.^24^ demonstrated cortisol excess to be associated with T‐cell depletion and anergy in ACC TME, while a recent immunotherapy clinical trial revealed a pattern of immune resistance among cortisol‐secreting ACC (CS‐ACC) tumours, with higher rates of immunotherapeutic failure among CS‐ACC patients compared to the patients with nonCS‐ACC.^13^, ^25^, ^26^, ^27^ In this study, we utilized The Cancer Genome Atlas (TCGA) ACC cohort^28^ to characterize the TME of ACC by comparing TME immunogenomic profiles of CS and nonCS‐ACCs. We have also investigated the correlations and prognostic significance of differentially expressed immune genes (DEIGs) and tumour‐infiltrating immune cell (TIIC) profiles.

## 2 METHODS

### 2.1 Data acquisition, patient demographics & tumour pathology

We utilized the RNA sequencing count table data of Adrenal Cortical Carcinomas (*N* = 92) from The Cancer Genome Atlas (TCGA) Firehose Legacy Cohort.^28^ Of the 92 patients in the TCGA cohort, 67 (73%) patients, with common type ACCs (non‐myxoid/non‐oncocytic), did not undergo neoadjuvant therapy and had reported hormone hypersecretion and mRNA expression values were included in the study cohort. The American Joint Commission on Cancer Staging Manual, 8th edition, was used to determine TNM classification. Categorical variables were presented as frequency and percentages and compared using chi‐square or Fisher's exact test, as appropriate. Continuous variables were reported as median values with interquartile range (IQR) and compared using the Kruskal–Wallis test.

### 2.2 Computational immunogenomic deconvolution

The Cancer Genome Atlas (TCGA, Firehose Legacy) was accessed through cBioPortal (https://www.cbioportal.org/). *CIBERSORTx* was used to estimate tumour‐infiltrating immune subsets (including B cells, CD4^+^T cells, CD8^+^T cells, dendritic cells, macrophages, natural killer cells and neutrophils). *CIBERSORTx* is a computational immunogenomic platform, a publicly available web‐based deconvolution program (https://cibersortx.stanford.edu)^29^. All genes with quantified mRNA expression (log RNA Seq V2 RSEM) in TCGA database (*n *> 19,000) were compared between CS‐ and nonCS‐ACCs. The significance criteria for DEG were set at a *p*‐value and *q*‐value < 0.05. After characterizing the relationships between DEGs and comparing the expression profiles between CS‐ACC and nonCS‐ACC TMEs, DEGs were categorized according to biological function using Panther Gene Classification.^30^ All DEIGs and TIIC associations were constructed in heatmap format to represent all potential associations and analysed in their relation to patient OS and DFS. Additionally, the mRNA expression of genes involved in steroid metabolism was analysed for their correlations with TIICs and prognostic DEIGs. Gene expression signatures were compiled by normalizing the sum of the gene mRNA Z‐scores (log RNA Seq V2 RSEM) relative to the median on a scale of −5 to 5. Patients with positive cumulative normalized expression levels (≥0.00) were assigned to the high signature expression group and those with negative cumulative normalized expression levels (<0.00) were assigned to the low signature expression group.

### 2.3 Patient outcomes analysis

Survival analysis was analysed by time‐to‐event Cox regression models for overall survival (OS) and disease‐free survival (DFS). OS was defined as the time from the date of index operation to the date of death. DFS was defined as the time from index operation to the date of documented disease recurrence or death. Kaplan‐Meier method and log‐rank test were used to compare OS and DFS of ACC patients according to mRNA expression signature profiles. Significance for OS and DFS analysis was set at a *p*‐value < 0.05.

### 2.4 Data availability

The Cancer Genome Atlas (TCGA, Firehose Legacy) was accessed through cBioPortal (https://www.cbioportal.org/) (https://www.cbioportal.org/study?id=605903f6e4b0242bd5d4433b).

### 2.5 Statistics

All quantitative comparison, correlation and survival analyses were performed using the 1.1.383 R statistics software (R Core Team Vienna).

## 3 RESULTS

### 3.1 Patient demographic, tumour pathology and treatment parameters of adrenocortical carcinoma

We identified 67 individuals in the TCGA ACC cohort with pathologically confirmed common type ACC with reported mRNA expression data and who did not undergo neoadjuvant therapy.^28^ Of these, 32 (47.8%) had CS‐ACC and 35 (52.2%) were nonCS‐ACC tumours. Excess cortisol secretion was diagnosed by biochemical assessment in 7 (21.9%) CS‐ACC patients (subclinical Cushing's syndrome) and by both clinical and biochemical assessment in 25 (78.1%) CS‐ACC patients (clinical Cushing's syndrome). The groups were similar in age at diagnosis (*p *= 0.37), race (*p *= 0.26), tumour stage T (*p *= 0.81), nodal status N (*p *= 0.14), metastasis M (*p* = 1.00) and clinical stage (*p *= 0.43). The CS‐ACC group was female‐predominant compared to the nonCS‐ACC group (81.2 vs. 45.7%, *p *< 0.01). CS‐ and nonCS‐ACC tumours demonstrated similar fractions of genome alteration (*p *= 0.97), mutation count (*p *= 0.193), mitotic count (*p *= 0.08) and rate (*p *= 0.72), tumour necrosis (*p *= 0.67), Weiss Score^31^ (*p *= 0.77) and rates of vascular invasion (*p *= 0.60). Both groups reported similar resection margins (*p *= 0.675) and underwent similar rates of adjuvant (*p *= 0.66) therapy, as well as mitotane (*p *= 0.12) and radiation therapy (*p *= 0.40). CS‐ACC patients experienced higher rates of ACC recurrence (62.5 vs. 31.2%, *p *= 0.02). Demographic, clinical and pathologic features of the study cohort by cortisol secretion are further summarized in Table S1.

Cortisol secretion was not significantly associated with shortened overall survival (OS) (hazard ratio [HR] 1.83; 95% confidence interval [CI] 0.82 – 4.07, *p *= 0.14) but was significantly associated with shortened disease‐free survival (DFS) (HR 2.34; 95% CI 1.13 – 4.85, *p *= 0.02). The 5‐year OS was 59.6% for nonCS‐ACCs and 51.6% for CS‐ACCs. The 5‐year DFS was 59.5% and 30.1% for nonCS‐ and CS‐ACC tumours respectively. The poor DFS prognosis associated with CS‐ACC despite similar patient demographics, tumour pathology and treatment protocols commonly associated with prognosis (cancer stage, Weiss Score, adjuvant therapy) is suggestive of a possible direct impact of cortisol secretion on ACC biology or TME immune opposition underlying patient DFS.

### 3.2 Differential Immunologic Gene mRNA Expression (DEGs) of cortisol‐secreting and non‐cortisol‐secreting adrenocortical carcinoma

**FIGURE 1:**
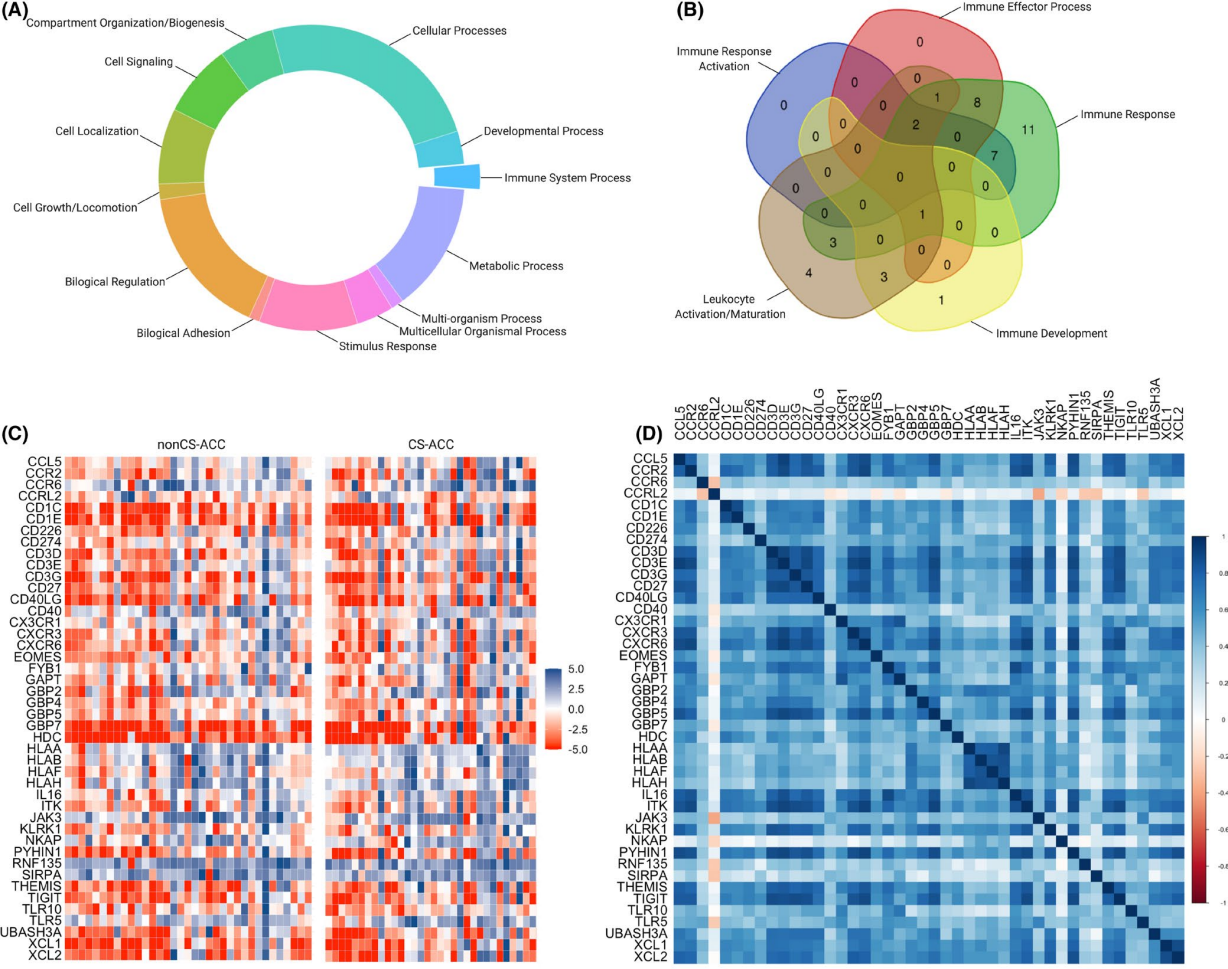
Differentially expressed immune genes (DEIGs) of adrenocortical carcinoma stratified by cortisol secretion. (A) Categorization of all differentially expressed genes (DEGs). (B) Subcategorization of DEGs directly involved in immunological processes (DEIGs). (C) Heat map of DEIGs between CS‐ACC and nonCS‐ACC. (D) Heat map of mRNA expression relationships between DEIGs

Analysis of all genes (*n* > 19,000) with quantified mRNA expression in TCGA database demonstrated 1,612 differentially expressed genes (DEGs) between CS‐ and nonCS‐ACC tumours. Of these DEGs, 1,021 were classifiable using Panther Genomic Classification (Figure 1A). Forty‐four (4.3%) genes of those classifiable were identified to be directly related to immunological processes and termed differentially expressed immune genes (DEIGs). On subcategorization of immunological processes using Panther Genomic grouping, DEIGs were primarily involved in immune response and leucocyte activation and maturation. The distribution of immunological processes is summarized in Figure 1B. Expression profiles of the 44 DEIGs stratified according to cortisol secretion and all mRNA expression intercorrelations represented in heatmap format are shown in Figure 1C. Forty‐three (97.7%) of the categorizable DEIGs identified showed decreased mRNA expression levels in CS‐ACC compared to nonCS‐ACC tumours. Uniquely, *CCRL2* showed elevated mRNA expression levels within CS‐ACC TME compared to nonCS‐ACC. Aside from *CCRL2*, all DEIGs showed positive mRNA expression correlations with one another (*r* ≥ 0.00), suggesting common or related transcription factors and/or cell processes, with CCRL2 as an exception (Figure 1C). *CCRL2* mRNA expression was negatively associated with that of several other DEIGs, including *CCR6* (*r* = −0.28), *JAK3* (*r* = −0.38), *NKAP* (*r* = −0.21), *RNF135* (*r* = −0.27), *SIRPA* (*r* = −0.27) and *TLR5* (*r *= −0.25) (Figure 1D). *CCRL2* mRNA expression was negatively associated with resting CD4 memory T cells (*r* = −29).

### 3.3 Tumour‐Infiltrating Immune Cell (TIIC) profiles of adrenocortical carcinoma

**FIGURE 2:**
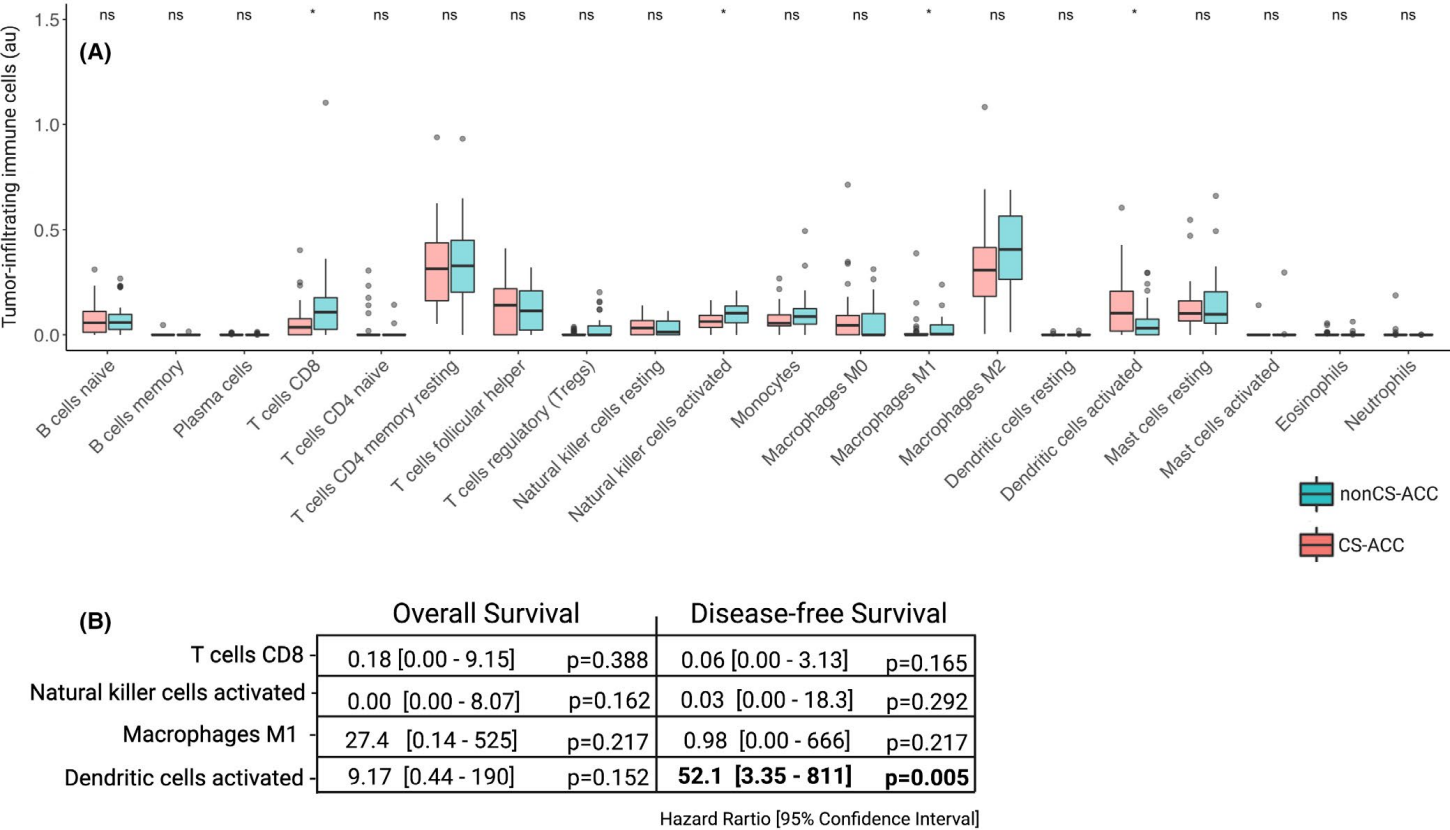
Tumour‐infiltrating immune cell (TIIC) profiles of adrenocortical carcinoma (ACC). (A) Scaled absolute value of tumour infiltration by immune cell types estimated by *CIBERSORTx* in ACC tumours and stratified into subgroups by cortisol secretion, abs, absolute arbitrary units; ns = *p*‐value ≥ 0.05; *p‐value < 0.05. (B) Impact of differentially expressed TIIC subtypes (CD8 T cells, activated natural killer cells, M1 macrophages, activated dendritic cells) on overall (OS) and disease‐free survival (DFS). Regression analysis, expressed as univariate Cox regression hazard ratio (HR) and 95% confidence interval (95% CI): HR [lower 95% CI – higher 95% CI], bold = *p*‐value < 0.05

The immunogenomic TME deconvolution using the *CIBERSORTx* platform elucidated a distinct TIIC landscape among CS‐ACC compared to nonCS‐ACC based absolute TIIC estimations (Figure 2A). Median proportional TIICs profiles for CS‐ and nonCS‐ACCs are shown in Figure 2A. CS‐ACC tumours demonstrated depletion of CD8^+^ T cells (*p *= 0.02), activated natural killer cells (NK_a_) (*p *= 0.04), as well as M1 macrophages (*p *= 0.04), and increased infiltration of activated dendritic cells (DC_a_) (*p *= 0.02). Of these four differentially infiltrated immune cell types that are identified in CS‐ and nonCS‐ACC TMEs, DC_a_ was the only TIIC population with significant prognostic association. Increased DC_a_ infiltration was the only TIIC population with significant prognostic association. Increased DC_a_ infiltration associated with poor DFS (HR 78.9, 95% CI 7.51 – 829, *p *< 0.01) (Figure 2B).

### 3.4 Steroid metabolism gene expression comparison and tumour‐infiltrating immune cells in cortisol‐secreting adrenocortical carcinoma

**FIGURE 3:**
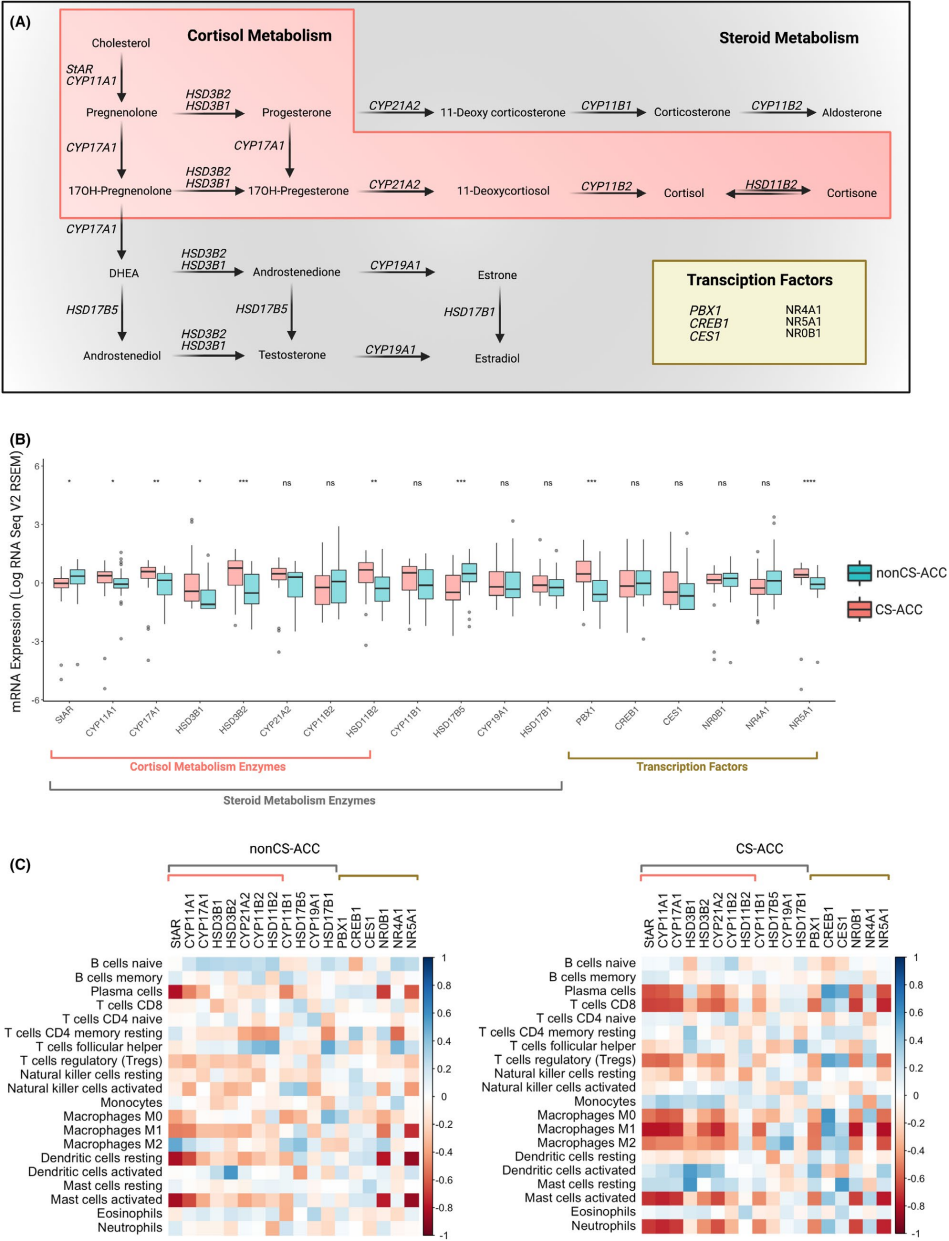
Steroid metabolism gene expression comparison by cortisol secretion and correlations to tumour‐infiltrating immune cells (TIICs). (A) Schematic of steroid metabolism enzyme and transcription factor gene expression. (B) Comparison of steroid metabolism gene expression by cortisol hypersecretion. (C) Heat map of steroid metabolism gene expression correlations with tumour‐infiltrating immune cell (TIIC) subtypes in nonCS‐ACC and CS‐ACC; ns = *p*‐value ≥ 0.05; *p‐value < 0.05, **p‐value < 0.01, ***p‐value < 0.001, ****p‐value < 0.0001

The mRNA expression of all genes underlying steroid metabolism enzymes, including cortisol synthesis, and influential transcription factors are depicted in Figure 3A. The mRNA expression levels of the steroid synthesis genes and transcriptions levels of ACC tumours are compared according to cortisol secretion in Figure 3B. CS‐ACCs demonstrated decreased mRNA expression of *StAR* and *HSD17B5* and increased mRNA expression of *CYP11A1*, *CYP17A1*, *HSD3B1*, *HSD3B2*, *HSD11B2*, *PBX1 and NR5A1* compared to nonCS‐ACC (*p*‐values < 0.05). Steroid metabolism gene expression correlations with TIIC subtypes among nonCS‐ACC and CS‐ACC are represented in heatmap format in Figure 3C,D. *StAR*, *NR0B1* and *NR5A1* mRNA expressions were negatively associated with activated mast cell in both nonCS‐ACC and CS‐ACC (*r* ≤ ‒0.70) in CS‐ and nonCS‐ACCs. Among CS‐ACC, the mRNA expression of genes coding for enzymes contributing to cortisol synthesis *StAR*, *CYP11A1*, *CYP17A1*, *HSD3B2* and *CYP21A2* and steroid metabolism transcription factors *NR0B1* and *NR5A1* were associated with decreased plasma B cell, CD8 T cell, M1 macrophage, activated mast cell and neutrophil infiltration (*r* ≤ ‒0.50).

### 3.5 Differential tumour immune cell infiltration, immune‐related differentially expressed genes patient outcomes in cortisol‐secreting adrenocortical carcinoma

**FIGURE 4:**
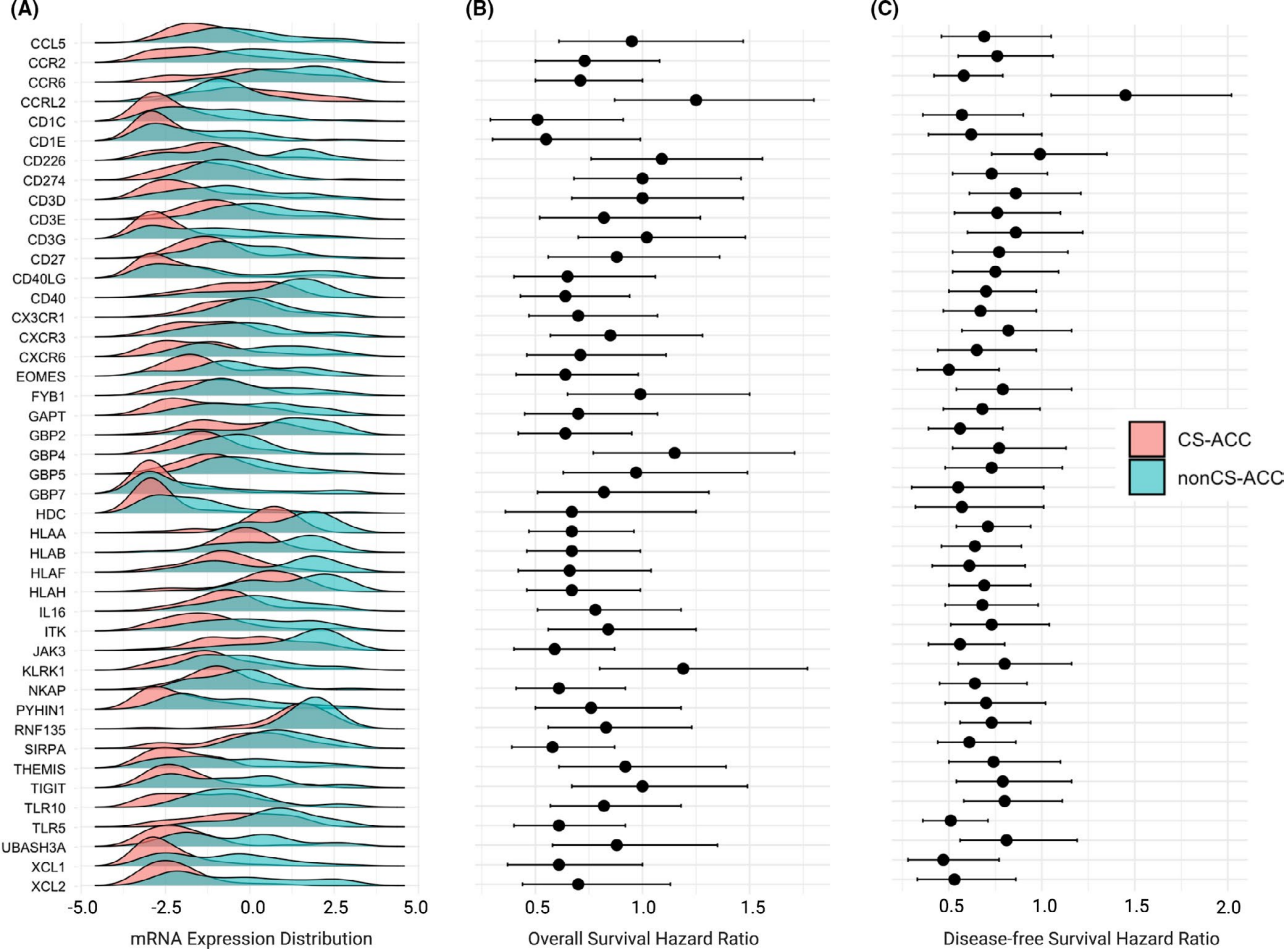
Differentially expressed immune genes (DEIGs) by cortisol secretion and associated patient outcomes in adrenocortical carcinoma (ACC). (A) The distribution of normalized mRNA expression of differentially expressed immune genes (DEIGs) in CS‐ACC and nonCS‐ACC tumours. (B) Overall survival (OS) univariate cox‐regression analysis according to gene mRNA expression. Regression analysis expressed as univariate Cox regression hazard ratio (HR) and 95% confidence interval (95% CI): HR [lower 95% CI – higher 95% CI]. (C) Disease‐free survival (DFS) univariate Cox regression analysis according to gene mRNA expression. Regression analysis, expressed as univariate Cox regression hazard ratio (HR) and 95% confidence interval (95% CI): HR [lower 95% CI – higher 95% CI]

The mRNA expression of 14 (31.8%) of the 44 DEIGs emerged as significant prognostic indicators (HR > 1.00, OS and DFS, *p *< 0.05) and made up the cumulative prognostic immune signature (*CCR6*, *CD1C*, *CD1E*, *CD40*, *EOMES*, *GBP2*, *HLAA*, *HLAB*, *HLAH*, *JAK3*, *NKAP*, *SIRPA*, *TLR5*, *XCL1)*. DEIGs contributing to this prognostic immune signature were suppressed in CS‐ACCs and can be grouped into several subcategories according to immune function, including chemokine and cytokine signalling (*CCR6*, *XCL1*), macrophage signalling (*GBP2*, *TLR5*), leucocyte antigen proteins (*HLA*‐*A*, *B*, *H*), T‐cell signalling (*CD1C*, *CD1E*, *EOMES*, *NKAP*), B‐cell signalling (*CD40*), DC signalling (*SIRPA*) and global immune development and response (*JAK3*) (Figure 4A–C). The distribution of DEIGs by cortisol secretion and the impact of DEIGs on OS and DFS are summarized in Figure 4 and Table S1. Nine DEIGs were positively associated with DFS only and included *CD40*, *CX3CR1*, *CXCR6*, *GAPT*, *HDC*, *HLAF*, *IL16*, *RNF135 and XCL2*. Functionally, these genes can be grouped into chemotactic signalling (*XCL2*), innate immune response (*GAPT*, *HDC*, *IL16*) and other (*RNF135*). The mRNA expression of *CCL5*, *CCR2*, *CD226*, *CD274*, *CD3D*, *CD3E*, *CD3G*, *CD27*, *CD40LG*, *CXCR3*, *FYB1*, *GBP4*, *GBP5*, *GBP7*, *ITK*, *KLRK1*, *PHYIN1*, *THEMIS*, *TIGIT*, *TLR10 and UBASH3A* showed no prognostic significance in DFS or OS (Figure 4A–C). There were no DEIG expression levels that were associated with OS and not DFS. The *CCRL2* gene mRNA expression was the only DEIG upregulated in CS‐ACC and was associated with poor DFS (HR 1.45, 95% CI 1.05 – 2.02, *p *= 0.03).

The *CCRL2* gene codes for the C‐C Motif Chemokine Receptor‐Like 2, a non‐signalling seven‐transmembrane domain receptor related to the atypical chemokine receptor (ACKR) family, however, and its role of this receptor in TME is elusive. ACKRs typically bind chemokines without G protein signalling activation to promote ligand internalization and degradation; however, more importantly, they regulate immune functions by scavenging chemokines from the local environment.^32^ Previous studies have demonstrated CCRL2 receptors to act as decoy receptors scavenging chemokines from the TME and their expression to be associated with poor dendritic cell trafficking.^33^ Elevated *CCRL2* expression has been shown in primary neutrophils relative to other immune cell types and further upregulated on neutrophil activation.^33^

Genes with mRNA expression found to be significantly associated with OS and DFS (*n* = 14, [*CCR6*, *CD1C*, *CD1E*, *CD40*, *EOMES*, *GBP2*, *HLAA*, *HLAB*, *HLAH*, *JAK3*, *NKAP*, *SIRPA*, *TLR5*, *XCL1*]*)* were compiled to create a composite immune mRNA expression signature characteristically suppressed in CS‐ACC tumours compared to nonCS‐ACC (Table S2). The bulk of the genes contributing to the prognostic mRNA signature downregulated in CS‐ACC were identified to code for interactive proteins crucial in the stepwise process of lymphocyte‐mediated.^34^ These steps include membrane and intercellular signalling proteins involved in T‐cell and NK cell activation (*CD1C*, *CD1E*, *NKAP*), recruitment (*CCR6*, *XCL1*), tumour recognition (*CD1C*, *CD1E*, *HLAA*, *HLAB*, *HLAH*) and CD8 T‐cell differentiation (*EOMES*).^35^, ^36^ Other gene products, including those of *GBP2* and *TLR5*, have been shown to contribute to the innate immune response through macrophage activation and enhanced phagocytic and oxidative killing.^37^, ^38^ Signal regulatory protein alpha (*SIRPA*) gene codes for the cell surface receptor for *CD47*. The *SIRPA*‐*CD47* has been shown to prevent the maturation of dendritic cells and promote immune tolerance of mature dendritic cells.^39^ Janus kinase (*JAK*) family of tyrosine kinases involved in cytokine receptor‐mediated intracellular signal transduction of the innate and adaptive immune system and mutations of this gene are characteristic of severe combined immunodeficiency.^40^

### 3.6 Prognostic differentially expressed immune gene and steroid metabolism gene correlations in adrenocortical carcinoma

**FIGURE 5:**
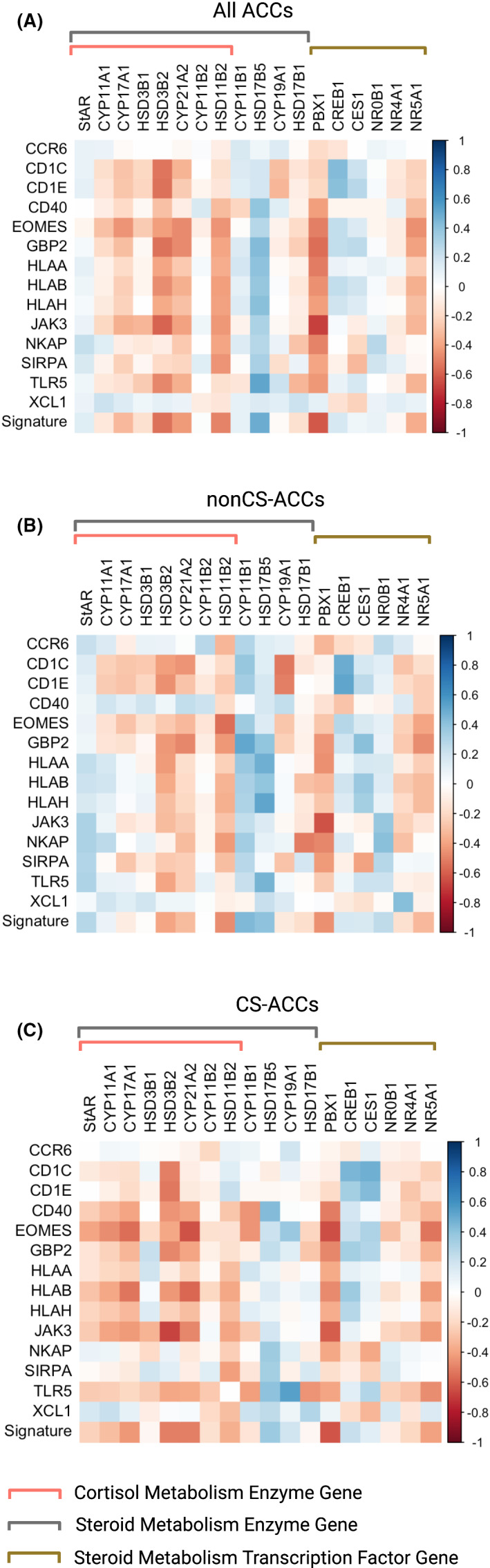
Expression correlations between prognostic differentially expressed immune genes (DEIGs) and steroid metabolism genes. (A) Heat map of prognostic differentially expressed immune genes (DEIGs) and steroid metabolism genes in all ACC patients. (B) Heat map of prognostic DEIGs and steroid metabolism genes in all nonCS‐ACC patients. (C) Heat map of prognostic DEIGs and steroid metabolism genes in CS‐ACC patients

Expression correlations between prognostic DEIGs and steroid metabolism genes are shown in Figure 5. The mRNA expression of genes coding for enzymes specific to cortisol metabolism (including *CYP11A1*, *CYP17A1*, *HSD3B1 HSD3B2*, *CYP21A2 and CYP11B2*) and transcription factors *PBX1* and *NR5A1* were negatively associated with the prognostic DEIGs. The roles of these genes in steroid metabolism can be found in Figure 3A. *PBX1* and *NR5A1* belong to the *PBX* homeobox and the nuclear receptor families of transcription factors respectively. *PBX1* and *NR5A1* govern the transcription of cortisol and sex hormone biosynthesis.^41^

### 3.7 Immunosuppressive signature of cortisol‐secreting adrenocortical carcinoma

**FIGURE 6:**
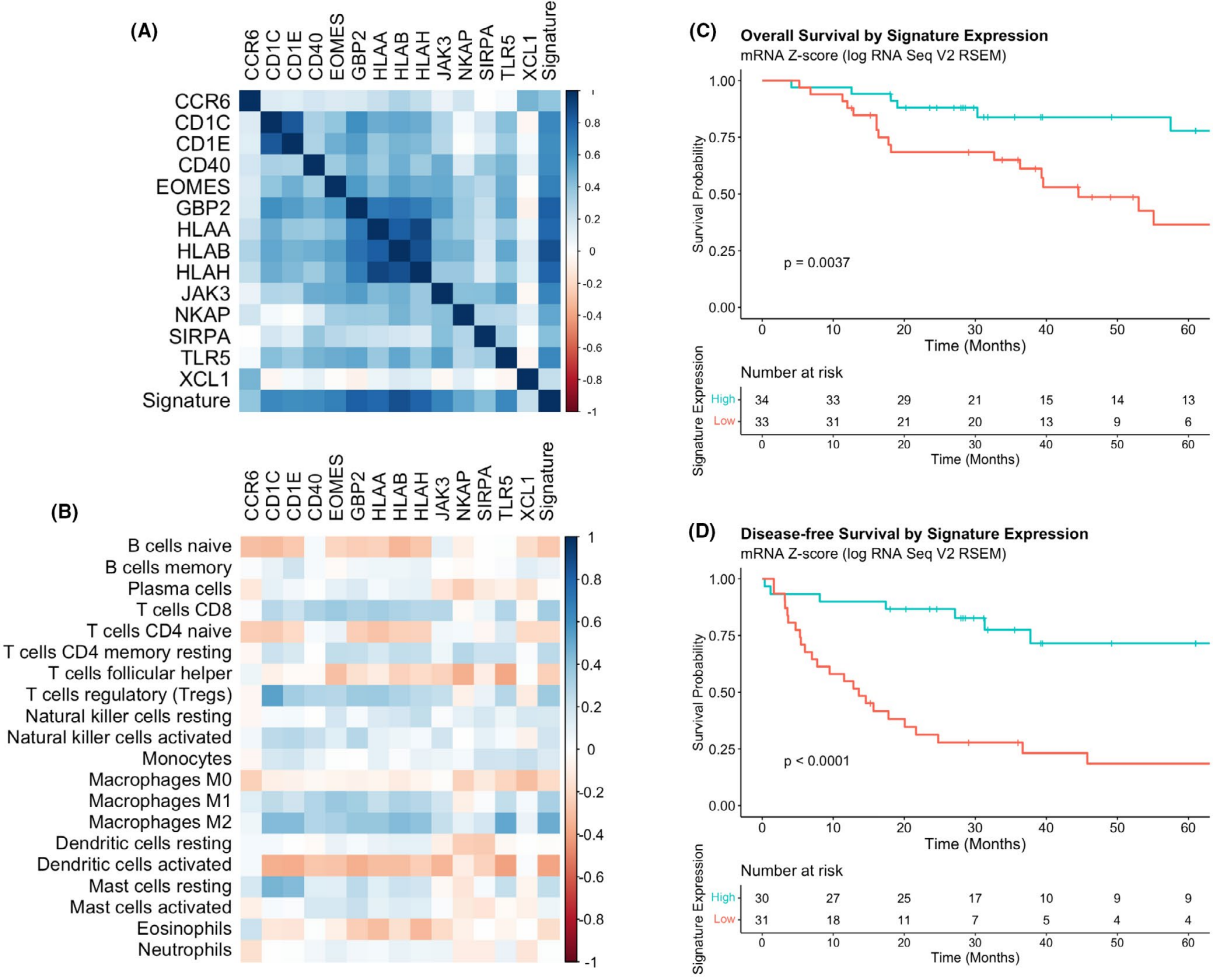
Immunosuppressive signature of cortisol‐secreting adrenocortical carcinoma (CS‐ACC). (A) Heat map of immunosuppressive signature of CS‐ACC with multiple intercorrelated gene mRNA expression sub‐clusters. (B) Heat map of individual gene expression contributing to CS‐ACC immunosuppressive signature and correlations with tumour‐infiltrating immune cell (TIIC) subtypes. (C) Overall survival (OS) comparing low and high expression of the total signature of ACC tumours. (D) Disease‐free survival (DFS) comparing low and high expression of the total signature of ACC tumours

Immunogenomic deconvolution of ACC TME revealed an immunosuppressive signature with multiple intercorrelated DEIG mRNA expression sub‐clusters. The HLA sub‐cluster (*HLA*‐*A*, *B*, *H*) (r ≥ 0.70) showed strong positive intercorrelations. Furthermore, *CD1C* and *CD1E* (*r* = 0.84) were found to be positively correlated (Figure 6A). The strong associations between the *HLA* mRNA expression values would also suggest a decreased MHC class I surface expression, which would result in decreased antigen presentation and T‐cell activation. Supportively, *HLA* sub‐cluster mRNA expression was positively associated with CD8^+^ T‐cell infiltration.

Cortisol‐secreting ACC tumours from patients with clinical and subclinical Cushing's syndrome showed significantly increased infiltration of CD8^+^T cells and resting mast cells in CS‐ACCs. Further sub‐analysis comparing the immunogenomic and TIIC profile was performed (Figure S1) to compare CS‐ACC patients diagnosed by biochemical and clinical evaluation (clinical Cushing's syndrome) with those diagnosed by biochemical evaluation alone (subclinical Cushing's ACC patients).

The relationships between the individual gene expression contributing to CS‐ACC signature showed many positive and negative correlations with TIICs (Figure 6B). Messenger RNA expression of *CD1C* and *CD1E*, a TCR contributory gene, was associated with T regulatory (T_reg_) cell infiltration (*r* > 0.35) and M2 macrophage (*r* > 0.43) tumour infiltration. TLR5 expression was positively associated with M2 macrophage infiltration (*r* = 0.51) and negatively associated with T follicular helper cells (*r *= −0.39) and DC_a_ (*r* = −0.41). Expression of the HLA cluster genes (*HLA*‐*A*,*B*,*H*) was positively associated with M2 macrophage infiltration (*r* > 0.39).

Lastly, the composite mRNA expression signature suppressed in CS‐ACCs was positively associated with CD8^+^ T cell (*r* = 0.35), T_reg_ cell (*r* = 0.36) and M2 macrophage (*r* = 0.49) infiltration and negatively associated with DC_a_ (*r* = −0.39) in the ACC TME, suggesting a link between the prognostic DEIG signature expression and prognostic TIIC profiles. Univariate Cox regression showed low expression of the mRNA signature was associated with significantly shorter OS (HR 3.43, 95% CI 1.42–8.28, *p *= 0.016) and DFS (HR 4.82; 95% CI 2.15–10.8, *p *< 0.001). The 5‐year OS for all ACC patients was 77.9% for the high expression group and 36.5% for the low expression group, while the DFS was 71.6% for the high expression group and 18.5% for the low expression group (Figure 6C,D).

## 4 DISCUSSION

In this study, we examined and defined the DEIGs and TIIC profiles of ACC tumour microenvironment and immunosuppressive signatures through computational immunogenomic deconvolution of the TCGA genomic data. Specifically, we noted differences between CS‐ACC and hormonally inactive or non‐cortisol‐producing hormonally active ACC tumours (nonCS‐ACC). Our findings strongly support previous studies where CS‐ACC was shown to be associated with immune resistant TME and poor patient outcomes compared to nonCS‐ACC despite similar pathology and stage.^24^ Furthermore, we demonstrated immunogenomic differences between CS‐ACC and nonCS‐ACC TME while identifying distinct mRNA expression profiles associated with immune process genes. The downregulation of many of these DEIGs was associated with poor patient outcomes and differential TIIC profiles. Consistent with a recent independent cohort study,^24^ CS‐ACC tumours demonstrated significantly lower levels of CD8 T cells compared to nonCS‐ACC. Additionally, CS‐ACC showed decreased infiltration of NK_a_ cells and M1 macrophages. DC_a_ tumour infiltration was observed to a greater degree in CS‐ACC tumours and associated with a poor DFS prognosis. These findings support the notion that excess cortisol secretion in the ACC TME may not only alter the TIIC abundance, diversity and activity, but also contribute to tumour immune escape, immunotherapeutic failure and adversely impact patient outcomes.

Cortisol‐secreting ACC tumours have been considered the more aggressive phenotype among ACC tumours. Despite the known immunosuppressive role of supra‐physiologic glucocorticoid levels, this is the first human study to characterize the immunogenomic associations of cortisol excess related to ACC TME and correlation to patient prognosis. It is understood that glucocorticoids play a key regulatory role in the cell transcription process and homeostasis. Previous studies have demonstrated major alterations in immune cell genome expression under the treatment of exogenous glucocorticoids.^42^, ^43^ In our study, about 1 in 20 of the genes showed significantly different expressions between CS‐ and nonCS‐ACC. Consistent with previous studies, DEGs were primarily related to cellular and metabolic processes and biological regulation; however, a small portion was identified to be directly related to immunological processes (DEIGs). This deductive analysis served as a starting point for our study to further define the potential immunosuppressive role of excess cortisol in the ACC TME.

Cortisol is a corticosteroid with both glucocorticoid and mineralocorticoid activity that is physiologically regulated by the hippocampus‐pituitary‐adrenal (HPA) axis. CS‐ACC tumours escape the HPA negative feedback loop, leading to cortisol concentrations often over threefold the upper limit of normal. Recent studies have characterized a variety of mechanisms by which excess cortisol and synthetic cortisol‐like therapeutics (prednisone, betamethasone, etc.) impair the immune response and effects of immunotherapy in various cancer types through immune cell deactivation, dampen immune cell recruitment and maturation as well as the induction of apoptosis in lymphocytes.^43^, ^44^, ^45^, ^46^ Supra‐physiologic doses of exogenous glucocorticoids are associated with poor immune checkpoint inhibitor (ICI) response, including programmed death (PD‐1) and PD‐1 ligand‐1 (PDL‐1) monoclonal antibodies and cytotoxic T lymphocyte‐associated antigen‐4 monoclonal antibodies.^44^, ^45^, ^46^ The mechanistic failure of ICIs in the setting of excess glucocorticoids has been mostly attributable to multimodal lymphocyte inhibition and deactivation.^42^, ^43^, ^44^, ^45^, ^46^ Importantly, however, glucocorticoids have been shown to regulate cytokine secretion in T/NK lymphocytes and potentiate the inhibitory capacity of programmed cell death 1 by upregulating its expression on T cells.^43^ In this study, cortisol secretion was associated with decreased CD8^+^ T and NK_a_ cell infiltration in the TME compared to nonCS‐ACC. This collage of evidence suggests combating glucocorticoid suppression of lymphocytes may serve as a potential therapeutic target worthy of investigation, particularly in CS‐ACC tumours.

Excess glucocorticoid signalling has also been shown to inhibit macrophage differentiation towards a proinflammatory phenotype by attenuating the induction of proinflammatory genes that inhibits their differentiation of M1 phenotype.^47^, ^48^, ^49^ In our study, we observed significant M2 macrophage infiltration to be the predominant macrophage phenotype in all ACC tumours. Significantly fewer activated M1 macrophages were noted within TME of CS‐ACC compared to nonCS‐ACC tumours. Increasing macrophage recruitment, maturation and activation may be another means of TME optimization and a potential avenue for future therapeutic development in CS‐ACCs.

Stimulation of the glucocorticoid receptor impacts NF‐κB family proteins to inhibit their transcriptional activity.^50^ This results in innate and adaptive immune suppression through the decreased expression of co‐stimulatory molecules, cytokines and chemokines as well as the upregulation of co‐inhibitory molecules.^49^, ^50^ As observed in this study, several downstream NF‐κB product genes showed downregulation in CS‐ACC (*CCR2*, *CD40*, *CD40LG*, *EOMES*, *TLR5*).^48^, ^49^, ^51^ Additionally, glucocorticoid‐mediated inhibition of NF‐κB signalling pathways has been shown to hinder DC maturation and antigen presentation efficiency.^51^, ^52^, ^53^

Although the use of the TCGA database empowered this study by providing a sufficiently robust database of clinicogenomic parameters to derive meaningful associations in characterizing the TME of these ultra‐rare tumours, the collaborative is limited to large, academic referral centres which may lead to selection bias towards more aggressive, later stage disease with over‐representation of CS‐ACC and metastatic disease. This potential selection bias may limit the generalizability of our conclusions. Furthermore, the collaborative nature of the TCGA database also limits the granularity of clinical data available. For example, the TCGA database only reports on ACC hormone hypersecretion (nonfunctional, cortisol, aldosterone, oestrogen etc.) and does not include the diagnostic test use or laboratory values. Our analysis was limited to utilizing clinical and biochemical evaluation of excess cortisol production as a surrogate for degree of Cushing's disease which showed a trend towards more severe immune suppressive immunogenomic and TIIC profiles but was limited by the low statistical power of sub‐analysis (Figure S1). Furthermore, the treatment of patients with ACC is very heterogeneous across institutions with variations in surgical technique, radiation therapy and mitotane regimen (including dose, frequency and therapeutic level). Altogether, such limitations hinder our ability to further characterize and account for many clinical and treatment factors that may impact OS and DFS in CS‐ and nonCS‐ACC patients. Additionally, our analysis is limited to bulk sample mRNA sample deconvolution using the CIBERSORTx algorithm.

Additionally, although the deductive design of this study benefits the sensitivity for identifying DEIGs between CS‐ and nonCS‐ACCs, this method, along with a relatively small patient population, may limit the specificity of our analysis, thus increasing the potential for type 2 errors and false positive correlations. Similarly, although there were no statistically significant differences in demographics, treatment and tumour stage/pathology between CS‐ and nonCS‐ACC patient groups, it is plausible that accumulation of factors more prevalent in the CS‐ACC group—but not statistically different—may conspire to negatively impact survival, potentially confounding the correlations identified in this study.

The CIBERSORTx algorithm for TIIC estimation is highly correlative for certain immune cell populations, including CD8 T cells and B‐cell subtypes; however, these methods are less precise at estimating DC populations and DC subtypes.^54^ Nonetheless, DC estimations were included in our analysis due to their crucial role in potentiating ICI and T‐cell activation. Although DC_a_ infiltration was increased in CS‐ compared to nonCS‐ACC and associated with shortened OS and DFS, we suspect a molecular process of DCs may be influenced by excess cortisol in the ACC TME that we are unable to investigate further with the available data (such as DC migration, maturation and antigen presentation efficiency). Mature activated DCs are equipped to capture antigens and to produce large numbers of immunogenic MHC‐peptide complexes to potentiate T‐cell immunity. However, glucocorticoids have been shown to distinctly alter the phenotype of DCs by stunting maturation, hindering migration and inhibiting the expression of MHC proteins.^51^, ^52^, ^53^ The overall impact of glucocorticoids on DCs has been summarized as a partial conversion to a monocyte‐macrophage phenotype and impaired capacity to reach maturation resulting in decreased T‐cell stimulation.^51^, ^52^ Altogether the impact of excess cortisol among CS‐ACC may result in increased accumulation of inefficient DC_a_ in the ACC TME.

This study may offer additional insight into why strong immune infiltration is rarely seen in CS‐ACC and why current immunological therapeutic options have been of limited efficacy. Our findings suggest that the ACC cortisol hypersecretion impacts TME in favour of immune resistance. Excess cortisol in the ACC TME may potentially facilitate more aggressive tumour biology and poor prognosis. To date, several studies have now highlighted the negative effects of synthetic glucocorticoids on the outcome of immunotherapy.^41^, ^42^ In line with this, patients with CS‐ACC were recently reported to experience decreased response to immunotherapy and experience poor patient outcomes compared to nonCS‐ACC patients when treated with anti‐PD‐L1 agent Pembrolizumab and mitotane.^25^

This study findings support two previous related but different studies. In 2004, Wolkersdörfer et al.^55^ suggested the immune escape of ACC may be the consequence of altered Fas/Fas‐L system expression and loss of MHC class H and HLA expression in an ACC cell line stimulated to secrete cortisol. In 2020, Landwehr et al.^24^ demonstrated decreased CD8^+^ T cell infiltration among CS‐ACCs compared to nonCS and CD8^+^ T cell infiltration to be associated with improved prognosis. The decreased expression of HLA‐A, B, F, H and CD8^+^ T cell infiltration among CS‐ACC and their associations with poor patient prognosis observed in this study would further support these findings and suggest a potential role for excess cortisol in impacting antigen presentation and lymphocyte activation in CS‐ACCs. Furthermore, the mRNA expression levels of several genes coding for cortisol synthesis enzymes (*CYP11A1*, *CYP17A1*, *CYP21A2)* and steroid metabolism transcription factors (*PDX1*, *NR5A1)* were upregulated in CS‐ACC and associated with decreased CD8^+^ T‐cell infiltration. These gene products and pathways may provide for actionable drug targets to combat immune resistance in CS‐ACCs.

In summary, our study characterized a distinct immunogenomic profile with a significant prognostic value associated with CS‐ACC compared to nonCS‐ACC that may contribute to the poor outcomes associated in patients with CS‐ACC. In depth future studies aimed at uncovering the full impact of excess glucocorticoid metabolism and secretion in TME and comprehensive targeting of steroid metabolism may provide new immunotherapeutic applications for effective treatment of aggressive and poorly responsive CS‐ACC tumours to improve patient survival. Our findings may help guide future studies needed to clarify the potential mechanisms of immune resistance and immunotherapy failure in CS‐ACC. Such insight may empower strategies to reduce the potentially harmful effects of excess cortisol secretion and synthetic glucocorticoids used to control side effects and symptoms associated with many immunotherapies.

## CONFLICT OF INTEREST

None.

## AUTHOR CONTRIBUTION

**Jordan J Baechle:** Conceptualization (lead); Data curation (lead); Formal analysis (lead); Methodology (lead); Visualization (lead); Writing‐original draft (lead); Writing‐review & editing (supporting). **David N Hanna:** Formal analysis (supporting); Visualization (supporting); Writing‐review & editing (supporting). **Sekhar Konjeti:** Formal analysis (supporting); Methodology (supporting); Visualization (supporting); Writing‐review & editing (equal). **Jeffrey C Rathmell:** Conceptualization (supporting); Supervision (supporting); Writing‐review & editing (supporting). **W Kimryn Rathmell:** Conceptualization (supporting); Supervision (supporting); Writing‐review & editing (supporting). **Naira Baregamian:** Conceptualization (supporting); Supervision (lead); Visualization (supporting); Writing‐original draft (supporting); Writing‐review & editing (lead).

## Supporting information

Figure S1

Table S1

Table S2
