# Supplementary figures and images for: Integrative computational immunogenomic profiling of cortisol‐secreting adrenocortical carcinoma
Source: J Cell Mol Med. 2021 Oct 19;25(21):10061–72. doi: 10.1111/jcmm.16936 (PMC8572764; doi:10.1111/jcmm.16936)

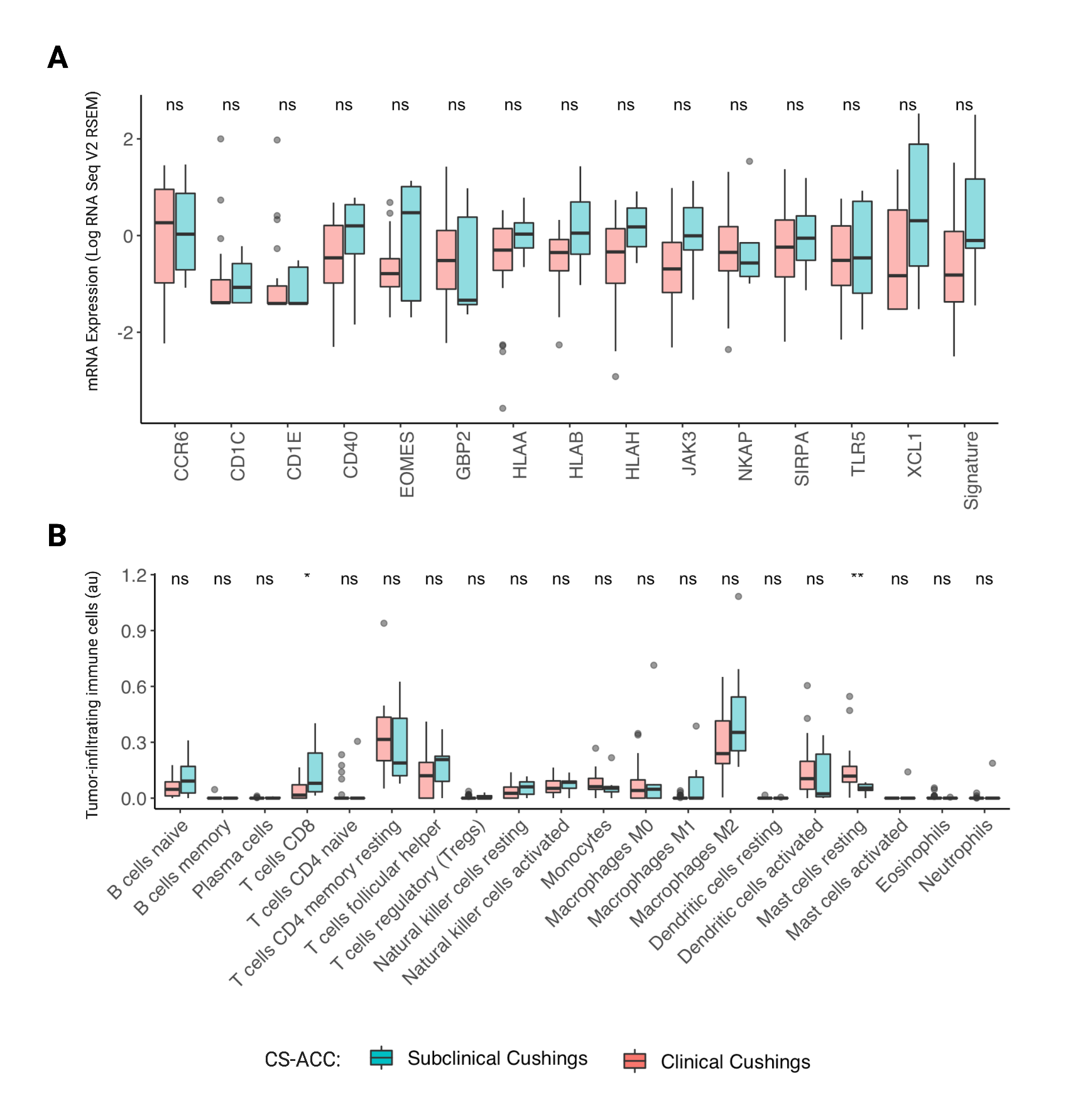

Supplement: Supplementary file 1 — Figure S1 [file JCMM-25-10061-s002.png]
