# Supplementary material for: Integrative computational immunogenomic profiling of cortisol‐secreting adrenocortical carcinoma
Source: J Cell Mol Med. 2021 Oct 19;25(21):10061–72. doi: 10.1111/jcmm.16936 (PMC8572764; doi:10.1111/jcmm.16936)
Supplement: Supplementary file 2 — Table S1 [file JCMM-25-10061-s001.docx]

**Supplemental Table 1. Patient Demographic, Tumor Pathology, and Treatment Parameters of Adrenocortical Carcinoma (ACC).** CS-ACC = cortisol-secreting adrenocortical carcinoma; nonCS-ACC = non-cortisol-secreting adrenocortical carcinoma; IQR = interquartile range; Age = age at diagnosis; Unknown = unreported data; HPF = high-power fields; RX = indeterminate margins.

|  | | **NonCS-ACC**  *N=35* | **CS-ACC**  *N=32* | **p-value** |
| --- | --- | --- | --- | --- |
| **Age (years) -Median [IQR]** | | 49.0 [36.0;56.0] | 47.5 [35.5;62.0] | 0.373 |
| **Sex** | Female | 16 (45.7%) | 26 (81.2%) | **0.006** |
|  | Male | 19 (54.3%) | 6 (18.8%) |  |
| **Race** | White | 28 (80.0%) | 29 (90.6%) | 0.262 |
|  | Asian | 1 (2.86%) | 0 (0.00%) |  |
|  | Black | 0 (0.00%) | 1 (3.12%) |  |
|  | Unknown | 6 (17.1%) | 2 (6.25%) |  |
| **Clinical Stage** | Stage I | 5 (14.3%) | 3 (9.38%) | 0.426 |
|  | Stage II | 17 (48.6%) | 12 (37.5%) |  |
|  | Stage III | 5 (14.3%) | 10 (31.2%) |  |
|  | Stage IV | 7 (20.0%) | 7 (21.9%) |  |
|  | Unknown | 1 (2.86%) | 0 (0.00%) |  |
| **T Stage** | T1 | 5 (14.3%) | 3 (9.38%) | 0.808 |
|  | T2 | 18 (51.4%) | 15 (46.9%) |  |
|  | T3 | 3 (8.57%) | 5 (15.6%) |  |
|  | T4 | 8 (22.9%) | 9 (28.1%) |  |
|  | Unknown | 1 (2.86%) | 0 (0.00%) |  |
| **N Stage** | N0 | 32 (91.4%) | 26 (81.2%) | 0.139 |
|  | N1 | 2 (5.71%) | 6 (18.8%) |  |
|  | Unknown | 1 (2.86%) | 0 (0.00%) |  |
| **M Stage** | M0 | 27 (77.1%) | 25 (78.1%) | 1.000 |
|  | M1 | 7 (20.0%) | 7 (21.9%) |  |
|  | Unknown | 1 (2.86%) | 0 (0.00%) |  |
| **Excess Hormone Secretion** | Cortisol | 0 (0.00%) | 15 (46.9%) | **<0.001** |
|  | Androgen + Cortisol | 0 (0.00%) | 16 (50.0%) |  |
|  | Androgen | 8 (22.9%) | 0 (0.00%) |  |
|  | Estrogen | 2 (5.71%) | 0 (0.00%) |  |
|  | Mineralocorticoids | 3 (8.57%) | 0 (0.00%) |  |
|  | Mineralocorticoid + Cortisol | 0 (0.00%) | 1 (3.12%) |  |
|  | None | 22 (62.9%) | 0 (0.00%) |  |
| **Laterality** | Left | 20 (57.1%) | 17 (53.1%) | 0.933 |
|  | Right | 15 (42.9%) | 15 (46.9%) |  |
| **Fraction of Genome Altered -Median [IQR]** | | 0.65 [0.39;0.87] | 0.49 [0.31;0.91] | 0.974 |
| **Mutation Count -Median [IQR]** | | 82.5 [69.2;106] | 93.0 [78.5;116] | 0.193 |
| **Mitoses Count -Median [IQR]** | | 5.50 [2.75;13.5] | 8.00 [5.50;23.0] | 0.080 |
| **Mitotic Rate** | < 5/50 HPF | 16 (45.7%) | 11 (34.4%) | 0.716 |
|  | > 5/50 HPF | 16 (45.7%) | 18 (56.2%) |  |
|  | Unknown | 3 (8.57%) | 3 (9.38%) |  |
| **Tumor Necrosis** | Absent | 9 (25.7%) | 6 (18.8%) | 0.670 |
|  | Present | 25 (71.4%) | 24 (75.0%) |  |
|  | Unknown | 1 (2.86%) | 2 (6.25%) |  |
| **Weiss Score -Median [IQR]** | | 6.00 [3.75;7.25] | 5.50 [3.75;7.25] | 0.774 |
| **Venous Invasion** | Absent | 20 (57.1%) | 14 (43.8%) | 0.597 |
|  | Present | 13 (37.1%) | 15 (46.9%) |  |
|  | Unknown | 2 (5.71%) | 3 (9.38%) |  |
| **Resection Margin** | R0 | 24 (68.6%) | 21 (65.6%) | 0.675 |
|  | R1 | 3 (8.57%) | 2 (6.25%) |  |
|  | R2 | 4 (11.4%) | 5 (15.6%) |  |
|  | RX | 2 (5.71%) | 4 (12.5%) |  |
|  | Unknown | 2 (5.71%) | 0 (0.00%) |  |
| **Adjuvant Therapy** | No | 27 (77.1%) | 23 (71.9%) | 0.657 |
|  | Yes | 6 (17.1%) | 8 (25.0%) |  |
|  | Unknown | 2 (5.71%) | 1 (3.12%) |  |
| **Mitotane Therapy** | No | 13 (37.1%) | 7 (21.9%) | 0.122 |
|  | Yes | 20 (57.1%) | 25 (78.1%) |  |
|  | Unknown | 2 (5.71%) | 0 (0.00%) |  |
| **New Neoplasm Following Initial Treatment** | No | 22 (62.9%) | 10 (31.2%) | **0.017** |
|  | Yes | 10 (28.6%) | 20 (62.5%) |  |
|  | Unknown | 3 (8.57%) | 2 (6.25%) |  |
