# Supplementary material for: Integrative computational immunogenomic profiling of cortisol‐secreting adrenocortical carcinoma
Source: J Cell Mol Med. 2021 Oct 19;25(21):10061–72. doi: 10.1111/jcmm.16936 (PMC8572764; doi:10.1111/jcmm.16936)
Supplement: Supplementary file 3 — Table S2 [file JCMM-25-10061-s003.docx]

**Supplemental Table 2. Impact of Prognostic Differentially Expressed Immune Genes (DEIGs) in Adrenocortical Carcinoma.** Univariant cox-regression analysis of DEIGs on overall survival (OS) and disease-free survival (DFS).

| DEIG | Overall Survival  Hazard Ratio [95% Confidence Interval] | p-value | Disease-Free Survival  Hazard Ratio [95% Confidence Interval] | p-value |
| --- | --- | --- | --- | --- |
| *CCR6* | 0.71 [0.50;1.01] | **0.048** | 0.58 [0.42;0.79] | **0.001** |
| *CD1C* | 0.51 [0.29;0.91] | **0.023** | 0.57 [0.36;0.90] | **0.015** |
| *CD1E* | 0.55 [0.30;0.99] | **0.044** | 0.62 [0.39;1.00] | **0.048** |
| *CD40* | 0.64 [0.43;0.94] | **0.023** | 0.70 [0.50;0.97] | **0.031** |
| *EOMES* | 0.64 [0.41;0.98] | **0.038** | 0.50 [0.33;0.77] | **0.001** |
| *GBP2* | 0.64 [0.42;0.95] | **0.028** | 0.56 [0.39;0.79] | **0.001** |
| *HLAA* | 0.67 [0.47;0.96] | **0.027** | 0.71 [0.54;0.94] | **0.018** |
| *HLAB* | 0.67 [0.46;0.99] | **0.046** | 0.64 [0.46;0.89] | **0.008** |
| *HLAH* | 0.67 [0.46;0.99] | **0.043** | 0.69 [0.50;0.94] | **0.02** |
| *JAK3* | 0.59 [0.40;0.87] | **0.008** | 0.56 [0.39;0.80] | **0.001** |
| *NKAP* | 0.61 [0.41;0.92] | **0.018** | 0.64 [0.45;0.92] | **0.014** |
| *SIRPA* | 0.58 [0.39;0.87] | **0.009** | 0.61 [0.44;0.86] | **0.005** |
| *TLR5* | 0.61 [0.40;0.92] | **0.019** | 0.51 [0.36;0.71] | **<0.001** |
| *XCL1* | 0.61 [0.37;1.00] | **0.042** | 0.47 [0.28;0.77] | **0.003** |
